# Review of the European Society for Photodynamic Therapy (Euro-PDT) Annual Congress 2020

**DOI:** 10.1684/ejd.2021.3973

**Published:** 2021-05-14

**Authors:** Colin A. Morton, Rolf-Markus Szeimies, Lasse R. Braathen

**Affiliations:** 1 Department of Dermatology, Stirling Community Hospital, Stirling, UK; 2 Department of Dermatology and Allergology, Klinikum Vest GmbH Academic, Teaching Hospital, Recklinghausen, Germany; 3 Private Office, Berne, Switzerland

**Keywords:** photodynamic therapy, non-melanoma skin cancer, actinic keratoses, biomarkers, dosimetry, daylight

## Abstract

This article reviews the 2020 European Society for Photodynamic Therapy (Euro-PDT) Annual Congress. Cutting edge studies included assessment of immunohistochemical variables influencing response of basal cell carcinomas and Bowen’s disease to PDT with p53, the only biomarker associated with good response in both conditions. A further study indicated that analysis of molecular markers, such as PIK3R1, could help select patients with actinic keratoses who demonstrate the best response to daylight PDT. Novel delivery protocols include artificial daylight, and laser-assisted and textile PDT. The meeting learnt of novel indications including antimicrobial PDT, as well as methods to optimise daylight PDT, including combination therapy for actinic keratoses. Adverse events were reviewed and options for painless and efficient PDT assessed, including the effect of reduced drug-light interval. A smartphone application was also evaluated which may be used to assist clinicians and patients in effective dosing and timing of daylight PDT via computational algorithms using data from earth observation satellites, to send light and ultraviolet dose information directly to patients’ smart phones.

Article accepted on 09/07/2020

The annual congress of Euro-PDT seeks to promote advances in research and in the clinical applications of photodynamic therapy (PDT) in dermatology. In the opening plenary session, Dr Morton (Stirling, UK) highlighted recent updates to guidelines on PDT by the European Dermatology Forum [1, 2] and the British Association of Dermatologists and Photobiology Group [3]. The European guideline confirmed the strongest 'A' recommendation based on the highest 'I' evidence level of efficacy of PDT for AK, Bowen's disease (BD) and superficial as well as thin nodular basal cell carcinoma (BCC) [1]. A 'BI' ranking was indicated for both the treatment and prevention of non-melanoma skin cancer (NMSC) in organ transplant recipients (OTR) and for the use of topical PDT in treating field cancerization. The guideline is unchanged regarding avoidance of PDT for invasive squamous cell carcinoma, but strong recommendations continue to encourage wider use of currently off-label indications for PDT in photorejuvenation, acne, onychomycosis and cutaneous leishmaniasis [1, 2]. The UK guideline, with links to clinical evidence summaries and evidence tables, summarised the evidence as strong 'offer' recommendations based on accumulated evidence for AK, BD (squamous cell carcinoma *in situ*), and superficial BCC, along with 'consider' recommendation for thin (<2 mm) nodular BCC and for field PDT as prophylaxis to reduce the emergence of new AK or non-melanoma skin cancer (NMSC) [3]. The presentation pointed participants to helpful Service Standards, accredited by NICE (National Institute for Health and Care Excellence) that describe all the elements required to provide a safe and effective topical PDT service [4].

## Non-melanoma skin cancer and PDT–basic aspects

Professor Gilaberte (Zaragoza, Spain) considered the role of biomarkers to help identify possible resistance of BCC and BD to topical PDT, noting treatment failure based on guidelines [1] in up to 25% (nBCC clearance at five years) and 30% (BD after two years of follow up), respectively. Her presentation highlighted the results of a retrospective study analysing clinical, histological and immuno-histochemical variables of 390 consecutive BCCs from 182 patients treated with PDT using methyl aminolaevulinic acid cream (MAL-PDT), with an overall clinical response rate of 83%, and mean follow-up time of 36 months [5]. Multivariate analysis revealed that the following variables were significantly associated with response to PDT: age, nodular subtype, presence of peritumoural inflammatory infiltrate, and p53 immunopositivity. Immunohistochemistry revealed positive p53 in 84.6% of responders, but only 15.4% with non-responsive tumours ($p = 0.011$). Tumours with increased immunostaining to β-catenin (expression linked with tumour aggressiveness) in the peripheral palisading of basal cell islands responded poorly to PDT ($p = 0.01$).
Based on a similar assessment of markers of resistance to MAL-PDT in BD, in which 33 lesions were treated (82% demonstrated a response at three months, dropping to 70% after six years), non-responders had larger lesions (mean

* This congress took place on 28th-29th February 2020 in Seville, Spain.

To cite this article: Morton CA, Szeimies RM, Braathen LR. Review of the European Society for Photodynamic Therapy (Euro-PDT) Annual Congress 2020. *Eur J Dermatol* 2021; 31(1): 17-21 doi:10.1684/ejd.2021.3973

of 2.5 cm compared to 1.5 cm for responders) [6]. Positive p53 immunostaining was observed in 90% of responders, but in only 30% of non-responders ($p = 001$). Cyclin D1 immunostaining was detected in 33% of responders vs. 80% of non-responders ($p = 0023$) and immunostaining of EGFR was intense in 14% of responders and 60% of non-responders ($p$= 0015). Based on a multivariate analysis, p53 was the only variable that significantly correlated with response to PDT, with a possible role in increasing protoporphyrin IX (PpIX) levels and subsequent cell death.

Dr. Fernández Nieto (Madrid, Spain) presented a prospective study of 22 patients, to analyse the clinical, histological and molecular response of patients with AK after treatment with daylight PDT (dPDT) and determine possible prognostic markers. Of the 22 patients, nine (40.9%) showed a poor clinical response to a single treatment. The degree of clinical improvement showed histological and immuno-histochemical correlation. The molecular study demonstrated overexpression of PIK3R1 in all cases with poor response, while cases with good response showed hypofunction of this gene. The ability to measure molecular markers such as PIK3R1 could be useful in patient selection for PDT.

Prof Mordon (Lille, France) discussed the hypothesis that ALAPDT and MALPDT may be used successfully with reduced or even without any drug-light interval. An important determinant affecting pain experienced by patients, shorter drug-light intervals are associated with reduced pain, mostly due to a continuous photodegradation of PpIX during its formation within lesions. He therefore confirmed that early protocols suggesting long incubation times of 14-18 hours with ALA-PDT were no longer appropriate based on a significant amount of evidence, reviewed in guidelines, indicating short incubation times to achieve equivalent efficacy but with reduced pain [1-3].

Dr Creusot (Plancenoit, Belgium) discussed the importance of managing and minimizing side effects. Pain is the key adverse event to discuss with patients. All clinical trials on daylight MAL-PDT show it to be significantly less painful than conventional PDT while being as efficacious for AK. Pain perception during PDT has been recently reviewed [7]. As expected from the nature of the PDT process, phototoxicity reactions occur and post-therapy sunlight protection remains important for up to 48 hours. Erythema, oedema, crusting and sterile pustules can be observed. Adverse effects of PDT, recently extensively reviewed and beyond pain, may be reduced by modifying the PDT technique, and the expected phototoxic reaction to PDT and severe sustained side effects are rare, reinforcing the role and importance of PDT as a safe and effective treatment option for certain skin conditions, in particular thin non-melanoma skin cancers and pre-cancerous sun damage [8].

## Daylight PDT (dPDT) for actinic keratoses (AK)

Professor Szeimies (Recklinghausen, Germany) sought to identify the audience's experience of dPDT, as the first presentation on a session dedicated to dPDT for AK. Using the mentimeter.com research tool (Mentimeter AB, Stockholm, Sweden), 82 conference participants took part in this survey. More than a half of them had over five years of experience in performing PDT, and dPDT was used routinely by 63 colleagues. All participants declared that they provided field-directed treatment (face/scalp) for mild-to-moderate AK, and 36% of all participants also declared that they provided lesion-directed dPDT in the same treatment area. The greatest beneficial effect dPDT offers is the lack of pain (8.7 based on a scale of 1-10), followed by convenience for the patient (8.2), ease of use (7.8) and fewer side effects compared to other field-directed therapies for AK (7.6). There was a high level of agreement regarding the following statements (rated from 1 to 5): repetitive use of dPDT offers good control of field-cancerized areas (4.1), MAL dPDT can be used in immuno-suppressed patients (transplant patients) with AK (4.1), and dPDT can be a powerful tool in reversing the signs of skin ageing (3.8). Later that day, the questionnaire was repeated and since the talks covered aspects of dPDT delivery, 25% of the participants became aware of the benefits of all-season dPDT based on the introduction of artificial light sources mimicking daylight exposure.

Dr. Eadie (Dundee, UK) presented novel research on SmartPDT®, a smartphone application to assist clinicians and patients in the delivery of dPDT. Accurate measurement of dPDT dose, in particular, with a minimum available dose for successful therapy, has proven to be difficult in routine patient care [9]. Rather than carrying a detector, earth observation satellites and computational algorithms send data on light and ultraviolet dose directly to the participant's smartphone. Under all weather conditions, agreement was established within 0.2% (±4.9%) for effective radiant exposure of protoporphyrin-IX between ground-based measurement and that determined by SmartPDT®. The ability of SmartPDT® to predict dPDT light dose 24 and 48 hours in advance of exposure was also assessed, and agreement with direct measurements within an average 21% and 17% was demonstrated, respectively.

Dr Arisi (Brescia, Italy) was unable to present in person, but kindly permitted sharing the outcome of a randomized split-face trial of conventional PDT (using an Aktilite CL128 [Galderma, Switzerland]) versus indoor dPDT (using a novel LED light source [Dermaris, Surgiris, France]) for multiple AK on the face and scalp in 24 patients. Conventional and indoor dPDT showed the same efficacy, but the latter was better tolerated by patients and resulted in lower inflammation scores. Dr Arisi reminds us that an outside temperature above 10 °C is recommended, with evidence that warmer temperatures are helpful; a 3% increase in clearance rates corresponds to a 5 °C increase in outdoor temperature [10].

Skin cancer nurse specialist, Louise Burns (Harrogate, UK) offered therapeutic tips and tricks, from a practical perspective, on performing dPDT. The need for, and importance of skin preparations was highlighted. Since dPDT is weather- and temperature-dependent, patient participation relative to different environmental scenarios is important.

## Combinations, modifications and new approaches

Dr Bedane (Limoges, France) presented a split-face comparison of daylight MAL-PDT (two-hour daylight exposure) versus PDT combined with ingenol mebutate for AK (ingenol mebutate for AK has now been withdrawn from the market). This study followed a comparison of dPDT versus blue-light PDT for AK performed by the same team, which confirmed similar high efficacy [11]. In the current study, Dr Bedane sought to improve on efficacy, in particular reducing recurrence rate. A pilot study was performed on 10 patients (average age of 76 years). The average number of AKs was 25, with Olsen Grade 1-2 before dPDT. Ten patients were evaluable at Month 1; the response rate was 87%. At Month 3, 10 patients were evaluable with a response rate of 86%. Eight of 10 patients presented with new AKs on the target zone. Ingenol mebutate was then randomly assigned to a hemi scalp and the first application was performed by the physician. The patient was educated to perform the second and third application in the following days, at home, on the same area. Ten patients were evaluable at Month 6, with a response rate of 88% for the ingenol mebutate-treated area and 74.4% for the untreated areas. Tolerance of ingenol mebutate application was considered satisfactory in 8/10, with prolonged redness and a sensation of burning only in two patients.

Dr Gelmetti (Brescia, Italy) was also unable to present at the meeting but submitted her presentation on the use of high-frequency ultrasound evaluation of the efficacy of PDT, ingenol mebutate and diclofenac 3% gel as treatment for multiple AKs and skin field of cancerization. Ninety patients with Olsen II AKs of the face and scalp were randomized to receive either MAL-PDT, ingenol mebutate 0.015% gel or diclofenac 3% gel. At baseline and three months after treatment, clinical and high-frequency ultrasound features were assessed on both treated lesions and surrounding photodamaged skin. MAL-PDT was the most effective treatment, showing the greatest reduction in cumulative AK area, resulting in greater reduction in SLEB thickness and an improvement of dermal and SLEB echogenicity.

These presentations highlighted the growing interest in combination therapies with PDT, summarized in a recent metanalysis [12]. Based on 10 randomized comparison trials, patients with AK, who were treated with combinations of PDT along with imiquimod cream, 5-fluorouracil cream, ingenol mebutate gel, tazarotene gel or calcipotriol ointment, showed overall higher response rates compared with monotherapy. Highest responses were observed for topical imiquimod cream, either prior to, or following PDT.

Dr Abi Rachid (Lille, France) presented a real-life evaluation of the treatment of AK based on textile PDT (fluxmedicare®). Randomized studies of flexitheralight® and Phosistos® have shown that textile PDT treatment is not inferior to conventional PDT, with AK complete clearance reaching 66-79% in the respective studies at three months, while offering excellent tolerance [13, 14]. The authors evaluated the efficacy and tolerance of this device in a real-life setting. Textile PDT had the same fluence and irradiance characteristics (12 J/cm$^2$, 1-2.6 mW/cm$^2$, 635 nm) as the Phosistos® device, but with some differences with regards to tissue conformation. Thirty-nine patients were treated, with a total of 417 AKs. With only one PDT session, the complete response rate was 72.6% at three months and 67% after six months of follow-up. The average maximal pain felt during the session was 0.9/10. This real-life study confirms the efficacy and excellent tolerance of textile PDT in the treatment of scalp AK.

Prof Mordon (also Lille, France) presented a study of 38 patients reflecting the use of artificial white LED light (Dermaris, Surgiris, Croix, France) PDT for AK [15]. Thirty-eight patients with Grade I-II AK of the scalp were treated after standard skin preparation, followed by 2 g of MAL cream (Metvixia, Galderma, France) applied to lesions and the surrounding normal skin. Immediately after cream application, the Dermaris was placed 20 cm from the scalp and switched on for 2.5 hours for photoactivation, leading to a total light dose of 26.1 J/cm, 2.9 mW/cm$^2$. Three months following treatment, the proportion of patients with less than five AK lesions was 68%. The median pain score was 0/10. Crusts, discomfort and pruritus were rated as mild or less in more than 87% of patients. Artificial dPDT using the white LED source was effective and almost painless, with minimal side effects for patients with AK lesions of the scalp.

Dr Braun (Ueberlingen, Germany) gave a memorable presentation reflecting 17 years of experience in practice of treating patients with MAL-PDT, including himself! He has steadily modified the protocol to optimize tolerance whilst maintaining efficacy. To achieve further improvements, in 2018, he combined the advantages of low-irradiance PDT and dPDT with further significant reduction in pain. Patients with AK and field cancerization received fractionated $CO_2$ laser pre-treatment, followed by MAL incubation under occlusion for 1.5-3 hours. Afterwards, patients were illuminated by Aktilite-LED (average dose: 4.8 J/cm$^2$; illumination time: 1:02 min; 74 mW/cm$^2$; with only cold air anaesthesia) and then subjected to one hour of daylight. In total, 136 cases (112 patients) with this PDT-light combination were treated during 2019, with clearance in 96.2% and average VAS score of 1.36.

## PDT beyond AK–a broader spectrum of indications

Dr Repelnig presented her team's experience of treating six challenging cases of digital Bowens and one patient with Bowenoid hyperplastic AK on one of their fingers for response to conventional MAL-PDT. The seven patients received a median of four sessions of PDT (2-10) within a median timeframe of five weeks (range: 1 to 75). BD showed a partial response in three patients, and no response in another three patients. Only one patient showed clearance of BD after two sessions of PDT, suggesting that monotherapy with MAL-PDT may not be sufficiently effective to eradicate extensive digital BD.

Professor Hofbauer (Zurich, Switzerland) discussed the role of PDT in the care of patients with organ transplants. Organ transplant recipients (OTRs) have a 50 to 100-fold increased risk of SCC and other skin cancers. For this high-risk population, close follow-up and early intervention to slow down cutaneous carcinogenesis is essential. As soon as skin cancers start developing, field-directed treatments or systemic medication should be considered. Field-directed treatments encompass 5-fluorouracil, imiquimod, diclofenac, ingenol mebutate and PDT, while most of these treatments are not officially registered for field use. PDT has been shown to be more effective than topical imiquimod in clearing AK in OTRs, with a superior three-month clearance rate (78% compared with 61%) after two treatment sessions [16]. Therapy-related skin reactions were more intense, but shorter in duration. Ablative fractional laser-assisted daylight photodynamic therapy (AFL-dPDT), compared to dPDT, conventional PDT (cPDT) and AFL alone, demonstrated an enhanced complete response with excellent tolerability relative to dPDT and cPDT for difficult-to-treat AK in OTRs [17].

Cyclic PDT with 5-aminolevulinic acid may reduce the incidence of SCC (invasive and *in-situ*) in OTRs, with a median reduction of 79.0% and 95.0% in 12- and 24-month post-treatment counts relative to pre-treatment counts at one month, respectively [18].

Mr. Todd (Dundee, UK) presented the results of a study to assess whether PDT outcomes for BD and superficial BCC are influenced by light dose [19]. The group compared the outcomes of two PDT light dose regimens, either 37 $Jcm^2$ or 75 $Jcm^2$. A total of 62 lesions (BD $n = 25$, BCC $n = 37$) were treated using a narrow-spectrum red light (630-635 nm); equal numbers with each regimen. Outcome was assessed three months after the first treatment, with a further treatment cycle administered if only partial response was achieved. Final outcomes were determined 12 months after initial treatment. At three months, 64.5% and 67.7% lesion clearance was achieved in the 37 $Jcm^2$ and 75 $Jcm^2$ groups, respectively. At 12 months, a striking difference was seen between the regimens, with 67.7% and 90.3% clearance in the 37 $Jcm^2$ and 75 $Jcm^2$ groups, respectively. The data indicates that higher irradiation doses may be more effective, and prospective studies are required to determine the optimal effective light dose. Although mathematical modelling shows that, compared with conventional broadband light, the currently used narrowband light, as used in this study, is more effective, with an *in vivo* study also indicating greater photobleaching, modelling of effective treatment depth is increased with the higher LED light dose.

Dr Ziane (Lille, France) presented initial results on the use of PDT with light-emitting fabrics for vulvar extramammary Paget's disease (EMPD) which affects the genital region. This can progress into invasive tumour and affects patients' quality of life. Surgery is the mainstay of treatment, but recurrences are common. Multiple clinical cases suggest that PDT could relieve patients' symptoms and control disease progression, but significant pain and the complex shape of the genital area limit the benefits of PDT for EMPD. Light-emitting fabric technology could address these issues, and a lighting device dedicated to vulvar and perianal areas has been developed. The PAGETEX® device is under investigation in an interventional clinical study (NCT03713203), which aims to determine whether MAL-PDT performed with PAGETEX® leads to disease control at three months. Only four patients are currently enrolled, however, a good response has been demonstrated in three based on the three-month follow-up period.

Dr Tim Maisch (Regensburg, Germany) provided a timely review, reflecting the reality of encountering infections that are resistant to current medication. Also discussed was the antimicrobial potential of the PDT process and the potentiation effect of PDT/combination therapy, all yet to be approved therapy protocols, but ripe for further research [2].

As an innovation for the congress, a round table discussion took place which considered which treatment was best for our PDT patients, with Professors R-M Szeimies, Gilaberte, Basset-Seguin and Morton as participants. Accepting the inevitable risk of bias from an audience providing evidence of PDT, the transformational effect of relatively pain-free dPDT over conventional red-light protocols, as a well-tolerated therapy for multiple AK/field cancerization presentations, was reaffirmed. Service logistics inevitably impact the order of prescription of therapies effective for AK, but PDT remains a strong choice where the service is supported.

Prior to his closing remarks, Prof Braathen, President of the Euro-PDT, announced the winners of the three poster prizes. The third prize was won by Dr Fonda-Pascual (Madrid, Spain) presenting a case series of four biopsy-proven cases of cutaneous Kaposi sarcoma treated by MAL-PDT every two weeks, with response after a mean of 3.5 sessions, optimized in two cases by pre-treatment with fractionated $CO_2$ laser. The second prize went to Dr Cerro Munoz (Zaragoza, Spain), who described a patient with nasal mucocutaneous leishmaniasis, on an anti-TNF-alpha biologic for rheumatoid arthritis, who had only partially responded to liposomal amphotericin B. Complete remission was achieved following three sessions of MAL-PDT with good tolerability and excellent cosmetic results. Dr Gracia-Cazana (Barbastro, Spain) was awarded first prize for her presentation on a retrospective study of patients receiving MAL-PDT treatment over a 10-year period, to compare histopathological findings between BCCs recurring after PDT against pre-treatment histology. Early treatment failure more often demonstrated aggressive subtypes with a high proportion of non-superficial treatment failure, probably resulting from misclassification with mixed subtypes, mostly observed in the 15 recurrent cases examined. All poster prize winners received a certificate and a financial reward.

The winners of the best oral presentations were Dr Mascaraque Checa (Zaragoza, Spain) (metformin as an adjuvant to PDT for NMS: an *in vitro* study demonstrating significant increase in tumour cell death using a combination of metformin and MAL-PDT) (first place), Dr Champaeu (Lille, France) (PDT for skin cancers: how to enhance 5-ALA penetration with polymeric-dissolving microneedles) (second place) and Dr Martin Braun (Uberlingen, Germany) (a new combined method of laser-assisted very-low-irradiance PDT with shortened daylight illumination) (third place).

Prof Braathen thanked the presenters for their excellent contributions and Paula Bloemen and Yves Hochedez for their excellent logistic services during and prior to the meeting, one of the last European medical meetings to take place before the COVID-19 pandemic impacted on the practice of all health care professionals. Prof Braathen praised the excellent and high scientific value of all contributions. Lots of new data were presented, outlining the continuing evolution of PDT to benefit as many patients as possible, with reduced adverse events in comparison with traditional therapy regimens. We hope to be able to meet again in 2021, as we anticipate further evidence to guide our selection of patients who are most likely to respond to PDT, increased knowledge of the merits of different methods of delivery of PDT, along with accumulating experience of the role of combination therapies, including PDT, in optimising patient care.

## Topical PDT in dermatology

### What's already known?

European evidence-based guidelines recommend PDT for certain superficial non-melanoma skin cancers and precancerous lesions.

Evidence is also strong for the efficacy of topical PDT for certain inflammatory and infectious disease indications, including photorejuvenation, acne, onychomycosis and cutaneous leishmaniasis.
dPDT offers a virtually pain-free alternative method of treatment that is widely adopted for AK.

## What's new

Over-expression of p53 may enhance the efficacy of PDT whilst over-expression of PIK3R1 may reduce efficacy.
Novel delivery protocols for PDT include artificial daylight and textile PDT.
A smartphone application can assist clinicians and patients in effective dosing and timing of dPDT, with light and ultraviolet dose information sent directly to patients' smart phones.

***Acknowledgements and disclosures.*** *Acknowledgements: the speakers mentioned in this article presented their data at the Euro-PDT Annual Congress 2020 and provided consent to inclusion of overviews of their presentations in this publication. The full text of each communication or poster can be found in the congress abstract book, available online at: http://euro-pdt.org/. Dr Morton, Prof Szeimies and Prof Braathen prepared this written article based on the presentations. Support: the authors received no support in the writing of this manuscript. Conflicts of interest: Dr Morton, Prof Szeimies and Prof Braathen received speaker fees and travel support from Galderma.*

**Open Access.** *This article is licensed under a Creative Commons Attribution 4.0 International License, which permits use, sharing, adaptation, distribution and reproduction in any medium or format, as long as you give appropriate credit to the original author(s) and the source, provide a link to the Creative Commons licence, and indicate if changes were made. The images or other third party material in this article are included in the article's Creative Commons licence, unless indicated otherwise in a credit line to the material. If material is not included in the article's Creative Commons licence and your intended use is not permitted by statutory regulation or exceeds the permitted use, you will need to obtain permission directly from the copyright holder. To view a copy of this licence, visit http://creativecommons.org/licenses/by/4.0/.*
